# Impact of 2004 Tsunami in the Islands of Indian Ocean: Lessons Learned

**DOI:** 10.1155/2011/920813

**Published:** 2011-05-18

**Authors:** Georges Ramalanjaona

**Affiliations:** ^1^Department of Emergency Medicine, St. John's Episcopal Hospital, 327 Beach 19th Street, Far Rockaway, New York, NY 11691, USA; ^2^Department of Emergency Medicine, St. John's Episcopal Hospital, 3264 Wolfson Dive, Baldwin, New York, NY 11510, USA

## Abstract

Tsunami of 2004, caused by a 9.0 magnitude earthquake, is the most devastating tsunami in modern times, affecting 18 countries in Southeast Asia and Southern Africa, killing more than 250,000 people in a single day, and leaving more than 1.7 million homeless. However, less reported, albeit real, is its impact in the islands of the Indian Ocean more than 1,000 miles away from its epicenter. This is the first peer-reviewed paper on the 2004 tsunami events specifically in the eleven nations bordering the Indian Ocean, as they constitute a region at risk, due to the presence of tectonic interactive plate, absence of a tsunami warning system in the Indian Ocean, and lack established communication network providing timely information to that region. Our paper has a dual objective: the first objective is to report the 2004 tsunami event in relation to the 11 nations bordering the Indian Ocean. The second one is to elaborate on lessons learned from it from national, regional, and international disaster management programs to prevent such devastating consequences of tsunami from occurring again in the future.

## 1. Introduction

Tsunami is a series of ocean waves typically caused by large undersea earthquakes or volcano eruptions at tectonic plate boundaries. These surges of water may reach 100 feet and cause widespread destruction when they crash ashore. They race across the sea at a speed up to 500 miles per hour and cross the entire Pacific Ocean in less than one day. Their long wavelength means that they lose very little energy along the way.

Tsunami of December 2004, caused by a 9.0 magnitude earthquake, is the most infamous tsunami of modern times with disastrous consequences in many areas [1]

- humanitarian toll: it affected more than 18 countries from Southeast Asia to Southern Africa, killing more than 250,000 people in a single day and leaving more than one million homeless,
- economic toll: it left several million of dollars of economic loss affecting fishing and tourist industries,
- environmental and medical threats including water pollution and flooding and endemic diseases.

The rationale for writing this paper is to report the tsunami events in the eleven nations bordering the Indian Ocean, as they received less publicity than their Southeast Asian countries counterpart although the 2004 tsunami had real humanitarian, economic, and environmental impact in these regions more than 1,000 miles away from the epicenter [2].

Furthermore, these regions are at risk from the devastating effects of future tsunami due to the presence of a tectonic interactive plate [3], absence of a tsunami warning system in the Indian Ocean, and lack of established communication network providing timely information to that region.

## 2. Methodology

This paper is a review of documents collected by WHO and other organizations/authors involved in disaster management during the 2004 tsunami.

## 3. Results and Discussion

### 3.1. Impact of Tsunami in the Islands of the Indian Ocean

These eleven countries bordering the Indian Ocean are Mauritius, Madagascar, Reunion Island, and Seychelles, Comoros islands and by geographical extension include countries in southern borders of Africa such as Somalia, Tanzania, Mozambique, and South Africa.

These individual countries suffered humanitarian loss with more than 3,000 people killed and left more than 10,000 homeless about 1,000 miles away from epicenter. In terms of economic toll, several million dollars were reported accompanied by environmental threat due to flooding.

Specifically included is a country by country report [4] with other south-Asian countries.

**(i) Mauritius**

Large waves completely submerged one village in north of the island. Although there was no death published, a significant economic loss in millions of dollars was reported.

**(ii) Madagascar**

Waves up to 10 meters were seen in southeastern region of the island. There was one death and more than 1,000 people homeless. Furthermore, there were considerable economic damages inflicted in touristic and fishing industries and infrastructure disruptions due to flooding and beach erosion (http://savannah.gatech.edu/cee/groups/tsunami/madagascar.html).

**(iii) Reunion Island**

It suffered mostly economic damages over one million dollars involving fishing industries with more than 200 boats sunk. No deaths were reported.

**(iv) Seychelles**

Ten people were reported killed, and flooding destroyed a major bridge between the capital Port Victoria and main airport. Also, the island reported devastating economic loss in millions of dollars due to hotels, housing, public utilities, and fishing damages.

**(v) Somalia**

More than 300 deaths were reported and 5,000 displaced.

**(vi) Tanzania**

Tsunami killed ten people with unknown number missing along with significant economic damages.

**(vii) Kenya**

Two deaths and two injured people were reported.

**(viii) South Africa**

8 people were killed about 8,000 km away from the epicenter.

**(ix) Indonesia**

122,232 deaths and 113,937 missing.

**(x) Shri Lanka**

30,974 killed and 4,698 missing.

**(xi) Thailand**

5,395 killed and 2,993 missing.

**(xii) Maldives**

82 deaths and 26 missing.

**(xiii) Malaysia**

68 deaths reported.

**(xiv) Myanmar**

59 killed.

**(xv) Bangladesh**

2 killed.

**(xvi) Burma**

90 killed.

### 3.2. Lessons Learned from 2004 Tsunami

To prevent the devastating effects of future tsunami, these islands of Indian Ocean have set their priorities in achieving 3 goals [5]:

- development of disaster tsunami program which include implementation of tsunami program at national level, regional, and international levels and coordination of all these programs,
- development of an Indian Ocean early warning system,
- development of tsunami research program.

#### 3.2.1. National Level

The most studied plans are the Madagascar plan, the tsunami early warning and response system in Mauritius, and the creation of the Department of Risk and Disaster Management in Seychelles.

**(i) Madagascar Plan**

It was developed in 2006 and is the most exhaustive of all the other national plans and should serve as a model for other islands. It includes 5 objectives:

- development of national evacuation plan on tsunami,
- establishment of early warning system in conjunction with regional system,
- increase public and community awareness through publication and training of media and local authorities,
- conduct mock exercises on tsunami,
- strengthen the operational capacity of national meteorological service to include national warning system.

#### 3.2.2. Regional Level

Disaster management is a regional priority in the Indian Ocean due to permanent threat of cyclones, floods, and tsunamis. The stated two goals set by a series of regional meetings in 2005 and 2006 are [6] the following:

- implementation of Indian Ocean tsunami warning and mitigation system (IOTWS), which focused on defining disaster management and reduction (prevention, mitigation, response and relief) of disaster by all the participating countries,
- development of integrated regional information network (IRIN) with the goals of creation of an early warning system for the islands in the Indian Ocean and ensuring adequate equipment to manage natural disasters including tsunamis.

The important issues are the cost of establishing such warning system in the Indian Ocean, the transfer of existing technology versus improving, old one, global warming and extreme weather events in that region.

#### 3.2.3. International Level

A series of international meetings have been convened to discuss the role international organizations [7]:

- international coordination meeting sponsored by UNESCO intergovernmental oceanographic commission in Mauritius in 2005 with a dual goal:
  - development of tsunami warning and mitigation system,
  - coordination of national tsunami warning center with regional centers,
- international strategy for disaster reduction (UN/ISDR) attended by representatives from the Indian Ocean countries and international experts on early warning system in 2006 with two objectives:
  - funding of projects and rehabilitation of roads and bridges
  - increase public awareness and training of key staff in tsunami preparedness and warning at all levels.

### 3.3. Future and Challenges

 The main challenge for all the islands of the Indian Ocean is to coordinate all the national efforts with existing regional and international endeavors to meet their stated priorities before the next tsunami events. 

The role of one special group of physicians should be mentioned at all these levels.

Emergency physicians are knowledgeable on the risks of tsunami and are trained in the field of disaster management, thus they are true expert. They should get involve as leaders in local, national, and international organizations as resources in disaster management as well as humanitarian institutions such as Red Cross.

## 4. Conclusion

This paper is the first peer-reviewed paper on the impact of the 2004 tsunami on the islands bordering the Indian Ocean and the lessons learned from this event from national, regional, and international organizations to prevent such events from occurring again in the future.

Tsunami is an ever-present and real threat for the these islands of the Indian Ocean due to the presence of a tectonic interactive plate.

Their disaster management priority is the development of an early tsunami warning system in order to effectively and timely communicate with all the people in that region. 

Disaster management should involve national, regional, and international organizations at all levels in order to develop tsunami program, fund tsunami projects, and continue research program.
